# Core and Accessory Genome Analysis of *Vibrio mimicus*

**DOI:** 10.3390/microorganisms9010191

**Published:** 2021-01-18

**Authors:** Iliana Guardiola-Avila, Leonor Sánchez-Busó, Evelia Acedo-Félix, Bruno Gomez-Gil, Manuel Zúñiga-Cabrera, Fernando González-Candelas, Lorena Noriega-Orozco

**Affiliations:** 1 Centro de Investigación en Alimentación y Desarrollo, A.C. (CIAD), Hermosillo, Sonora 83304, Mexico; qb_iliana@yahoo.com (I.G.-A.); lnoriega@ciad.mx (E.A.-F.); 2 Genomics and Health Area, Foundation for the Promotion of Health and Biomedical Research in the Valencian Community (FISABIO-Public Health), 46020 Valencia, Spain; sanchez_leobus@gva.es; 3 Centro de Investigación en Alimentación y Desarrollo, A.C. (CIAD) Mazatlán, Unit for Aquaculture and Environmental Management, Mazatlan, Sinaloa 82112, Mexico; bruno@ciad.mx; 4 Instituto de Agroquímica y Tecnología de Alimentos (IATA-CSSIC), 46980 Paterna, Spain; btcman@iata.csic.es; 5 Joint Research Unit Infección y Salud Pública, FISABIO-Universitat de Valencia, I2SysBio, CIBERESP, 46980 Valencia, Spain; fernando.gonzalez@uv.es; 6 Guaymas Unit, Centro de Investigación en Alimentación y Desarrollo (CIAD), Guaymas, Sonora 85480, Mexico

**Keywords:** *V. mimicus*, pan-genome, core genome, accessory genome, virulence genes, *V. cholerae*

## Abstract

*Vibrio mimicus* is an emerging pathogen, mainly associated with contaminated seafood consumption. However, little is known about its evolution, biodiversity, and pathogenic potential. This study analyzes the pan-, core, and accessory genomes of nine *V. mimicus* strains. The core genome yielded 2424 genes in chromosome I (ChI) and 822 genes in chromosome II (ChII), with an accessory genome comprising an average of 10.9% of the whole genome for ChI and 29% for ChII. Core genome phylogenetic trees were obtained, and *V. mimicus* ATCC-33654 strain was the closest to the outgroup in both chromosomes. Additionally, a phylogenetic study of eight conserved genes (*fts*Z, *gap*A, *gyr*B, *topA*, *rpo*A, *rec*A, *mre*B, and *pyr*H), including *Vibrio cholerae*, *Vibrio parilis*, *Vibrio metoecus*, and *Vibrio caribbenthicus*, clearly showed clade differentiation. The main virulence genes found in ChI corresponded with type I secretion proteins, extracellular components, flagellar proteins, and potential regulators, while, in ChII, the main categories were type-I secretion proteins, chemotaxis proteins, and antibiotic resistance proteins. The accessory genome was characterized by the presence of mobile elements and toxin encoding genes in both chromosomes. Based on the genome atlas, it was possible to characterize differential regions between strains. The pan-genome of *V. mimicus* encompassed 3539 genes for ChI and 2355 genes for ChII. These results give us an insight into the virulence and gene content of *V. mimicus*, as well as constitute the first approach to its diversity.

## 1. Introduction

The genus *Vibrio* contains more than 100 species typically isolated from aquatic environments, and several of them may cause infections in humans and animals [1,2]. *Vibrio mimicus* has been recognized as an emergent pathogen in human diseases, and it has been isolated from cases of gastroenteritis, ear infections, and severe cholera-like diarrhoea, as well as from several marine products, aquatic plants, sediments, and water (marine, brackish, and freshwater) [3,4,5]. *V. mimicus* strains encode a wide variety of virulence factors, such as he, hemagglutinins, pili, metalloproteases, enterotoxins, and siderophores, which are mediated by several different mechanisms [5,6,7,8]. Moreover, studies of comparative genomics of *Vibrio* species have shown that genetic exchange among *Vibrionaceae* family species is a continuous process [6,9,10,11,12].

*V. mimicus* was first described as an atypical non-pathogenic strain of *Vibrio cholerae*; now, both are considered closely related species and share almost 64% of their genome [13]. Wang et al. [14] studied the genome mutations responsible for the biochemical metabolism differentiation between *V. mimicus* and *V. cholerae*. Those biochemical differences are due mainly by gene deletion. Additionally, *V. mimicus* has been used in comparative analyses of the core and pan-genome of *V. cholerae*, sharing approximately eight core genes from the superintegron (SI) [15,16]. *V. mimicus* and *V. cholerae* share many phenotypic characteristics but also virulence genes, such as cholera toxin (Ctx), toxin-coregulated pilus, and pathogenicity islands, among others [13]. This makes *V. cholerae* an appropriate outgroup when conducting evolutionary studies of *V. mimicus* [3,9,17]. Recently, the comparative genome analysis of *Vibrio metoecus* (RC341) and *Vibrio parilis* (RC586), previously characterized as environmental non-pathogenic variants of *V. cholerae*, demonstrated that *V. metoecus* evolved from *V. cholera/V. mimicus* lineages, while *V. parilis* from an ancestral *V. mimicus* lineage [16,18,19]. This type of information contributes to the understanding of *Vibrio* species diversity [10].

Several virulence genes typical of pathogenic *Vibrio* species have been found in the genome of *V. mimicus* strains. Nevertheless, no differences in the virulence potential have been found between environmental and clinical strains of *V. mimicus* [6,20,21]. It has been reported that these virulence genes could have an important role in *V. mimicus* adaptation to their natural environment [6], where pathogenic strains could emerge.

Several comparative genomic studies have attempted to explain the evolutionary history of *Vibrio* species. For instance, Lin et al. [22] studied twenty *Vibrio* genomes, suggesting a high variation as a response to its adaptation to the environment. Thompson et al. [9] analyzed 17 *Vibrio* species with Comparative Microbial Genomics (CMG) biotools [23] and obtained a pan-genome of approximately 26,504 genes. This study included 18 strains of *V. cholerae* (pan-genome = 6923 genes) and two strains of *V. mimicus* (pan-genome = 8306 genes). Lilburn et al. [10] also studied 11 *Vibrionaceae* family genomes, but the *V. mimicus* genome was not included. A pan-genome of 51,517 genes was reported, where 49,588 corresponded to CDS (coding DNA sequence).

Molecular phylogenetic has been used for decades to elucidate the relationships between species. Today, the wide availability of genomes facilitates the study of most pathogenic bacteria [24,25,26]. At the time of this study, there were about 4000 complete genomes of *Vibrio* species with 65% being of *V. cholerae* and *Vibrio parahaemolyticus*, while only 0.5% corresponded to *V. mimicus* (https://www.ncbi.nlm.nih.gov/genome/browse#!/prokaryotes/).

Despite the importance of *V. mimicus* as a human pathogen, little is known about its evolution and biology. The availability of genomic sequences of several *V. mimicus* strains makes it possible to study the evolution and pathogenic potential of this species. In this study, nine genomes of *V. mimicus* were analyzed. The pan-, core-, and accessory genomes were determined to characterize the virulence and biology of *V. mimicus*; in order to document their diversity and obtain the first outline of their pan-genome. In addition, a multilocus sequence analysis (MLSA) of eight housekeeping genes was performed to obtain information regarding their variability.

## 2. Materials and Methods

### 2.1. Bacterial Genomes

Nine genomes of *V. mimicus* were analyzed in this study. The genomes of *V. mimicus* CAIM-602^T^ (Vm602, PRJNA179483) [7], *V. mimicus* CAIM-1882 (Vm1882, PRJNA219179), *V. mimicus* CAIM-1883 (Vm1883, PRJNA219181) [20], and *V. mimicus* ATCC-33654 (Vm 33654, PRJNA231624) were sequenced by our research group, as previously reported [7,20]. The remaining five genomes sequences were obtained from the database of the National Center for Biotechnology Information (NCBI): *V. mimicus* MB451 (Vm451, PRJNA40509), *V. mimicus* VM223 (Vm223, PRJNA40483), *V. mimicus* VM603 (Vm603, PRJNA40241), *V. mimicus* VM573 (Vm573, PRJNA40243), and *V. mimicus* SX-4 (Vm SX4, PRJNA47421) [6,10,14]. Additionally, two *V. cholerae* genomes were downloaded and used as outgroups, *V. cholerae* O1 biovar El Tor str. N16961 (Vc 16961, PRJNA36) [27] and *V. cholerae* O395 (Vc 0395, PRJNA32853) [28]. Original contigs were annotated by RAST (Rapid Annotations using Subsystems Technology) [29] (http://rast.nmpdr.org) and NCBI (http://www.ncbi.nlm.nih.gov).

### 2.2. Generation of a Coding Core Genome

A genome-wide assembly and contig synteny were constructed with Mauve Genome Alignment software ver. 2.3.1 (Sydney, Australia) [30] using *V. mimicus* MB451 as a reference strain [6]. The contigs were assembled with Geneious R6 ver. 6.0.3 (Biomatters Ltd. Auckland, New Zealand) to obtain the two chromosomes (ChI and ChII) of all *V. mimicus* strains [20]. The nine assembled sequences of each chromosome of *V. mimicus* strains and their corresponding counterparts of *V. cholerae* strains were aligned using progressiveMAUVE ver. 2.3.1 (Sydney Australia) [31], and the output was transformed into a plain FASTA format alignment using a Perl script [32]. The genome of *V. mimicus* strain MB451 was used as a reference [6], and all the positions from the alignment that were not shared with this strain were removed from the core genome analysis. Annotation files from NCBI were processed using R [33] to detect the coordinates of the genes in the reference genome, and to determine the core genes or those highly conserved genes that were present in all strains [26,34]. Core genes were defined as genes that shared significant homology on at least 80% with the corresponding reference gene. The core genomes of both chromosomes were built by concatenating the FASTA files of the corresponding aligned core genes [32].

### 2.3. Phylogenetic Reconstruction

Phylogenetic reconstructions of the core genome of each chromosome were performed using Maximum Likelihood (ML). The ML phylogenetic tree was obtained using RAxML (Randomized Axelerated Maximum Likelihood v7.2.7) (Heidelberg, Germany) [35], applying the GTRGAMMA model of nucleotide substitution and 1000 bootstrap replicates. *V. cholerae* was used as an outgroup for this analysis.

### 2.4. Analysis of the Accessory Genome

A matrix of presence-absence genes of the accessory genome was created for each chromosome and clustered using the heatmap.2 function of the gplots R package (clustering method hclust) [36].

### 2.5. Comparative Microbial Genomics (CMG)

The nine genomes of *V. mimicus* were analyzed by the CMG-Biotools (Lyngby, Denmark) [23] to obtain a genome BLAST atlas of each chromosome using *V. mimicus* 451 as a reference genome.

### 2.6. Pan-genome Atlas

In addition, twenty-one genomes of *V. mimicus* uploaded until September 2019 were used to obtain the pan-genome atlas and the Average Nucleotide Identity (ANI) by Anvi’o ver.6.1 (http://merenlab.org/2016/11/08/pangenomics-v2/) [37]. The genomes analyzed were the nine genomes previously described plus twelve new genomes obtained from the NCBI: *V. mimicus* FDAARGOS113 (PRJNA231221), *V. mimicus* FDAARGOS112 (PRJNA231221), *V. mimicus* 523-80 (PRJNA242443), *V. mimicus* NCTC11435 (PRJEB6403), *V. mimicus* N2733 (PRJNA548872), *V. mimicus* N2763 (PRJNA548872), *V. mimicus* N2781 (PRJNA548872), *V. mimicus* N2789 (PRJNA548872), *V. mimicus* N2790 (PRJNA548872), *V. mimicus* N2810 (PRJNA548872), *V. mimicus* N2816 (PRJNA548872), and *V. mimicus* SCCF01 (PRJNA327733).

### 2.7. Multilocus Sequence Analysis (MLSA)

The sequences of eight housekeeping genes, *fts*Z (1200 nt), *gap*A (991 nt), *gyr*B (2065 nt), *top*A (1788 nt), *rpo*A (993 nt), *rec*A (1041 nt), *mre*B (1044 nt), and *pyr*H (732 nt), were obtained from the nine genomes of *V. mimicus* and the genomes of *V. cholerae* 16961, *V. parilis* (RC586), *V. metoecus (*RC341) [16,19] and *Vibrio caribbeanicus* ATCC-BAA-2122 [38], which were downloaded from NCBI. The nucleotide sequences were aligned with the MUSCLE program implemented in Geneious R6 ver. 4.8.5 (Biomatters Ltd.) with UPGMB (unweighted pair group method with arithmetic mean) as the clustering method (kmer4_6 as a distance measure for iteration 1 and pctid_kimura as a distance measure for iterations 2). An ML phylogenetic tree was created using RAxML by CIPRES Science Gateway [39], and the robustness of the topology was checked by 1000 bootstrap replicates. The consensus tree was elaborated by the 95% majority-rule of the replicates using DendroPy [40], and the edition of the phylogenetic tree was performed with the FigTree figure drawing tool (Ver 1.4.4), where *V. caribbeanicus* ATCC-BAA-2122 was used as an outgroup.

## 3. Results

### 3.1. Pan-Genome of V. mimicus

The general characteristics of the nine genomes of *V. mimicus* used in this study are shown in Table 1. The estimated size of Chromosome I (ChI) ranged between 2.82 and 3.05 Mbp with an average of 46.6% GC (guanine-cytosine content) and 2689 CDS were detected, whereas, in Chromosome II (ChII), the size ranged between 1.11 and 1.46 Mbp with an average of 46.0% GC, and 1164 CDS were identified. Additionally, according to the RAST server, the annotation was classified in several subsystems, reaching 436 in ChI and 132 in ChII (Table 1). The analysis of the pan-genome resulted in 3539 genes for ChI and 2355 genes for ChII.

**Figure 1:**
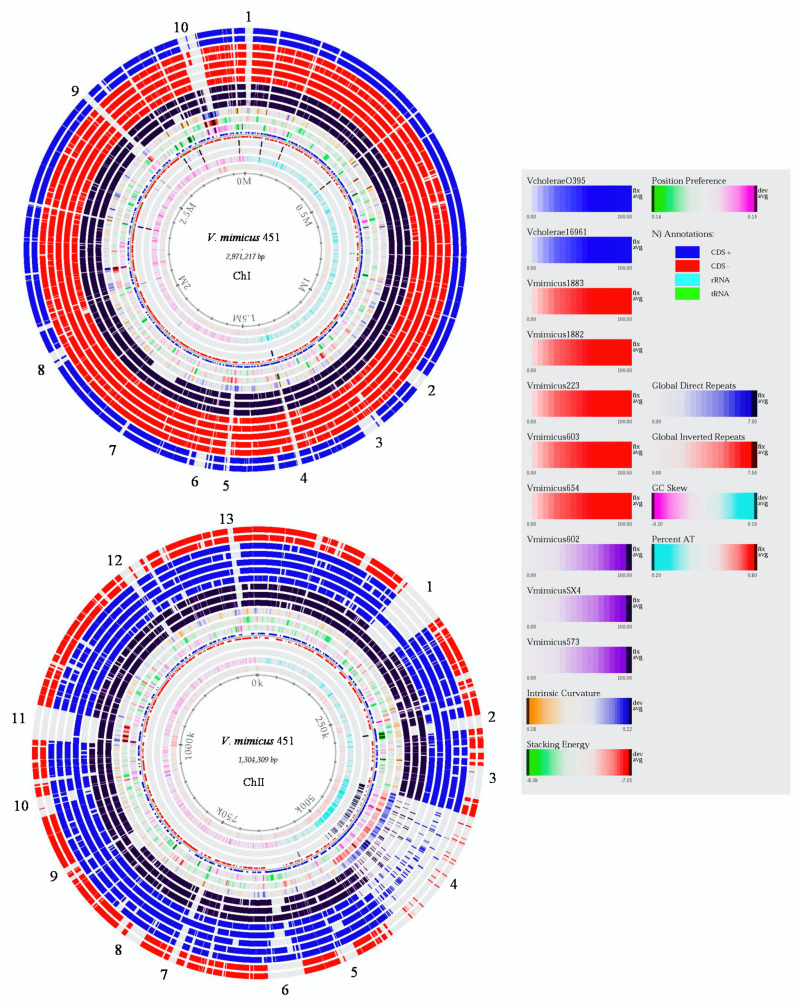
Genome BLAST atlas of both chromosomes of *V. mimicus* using *V. mimicus* MB451 as a reference. Order of the genomes from the inner dark circle: *V. mimicus* VM573, *V. mimicus* SX-4, *V. mimicus* CAIM-602^T^, *V. mimicus* ATCC-33654, *V. mimicus* VM603, *V. mimicus* VM223, *V. mimicus* CAIM-1882, *V. mimicus* CAIM-1883, *V. cholerae* 16961, and *V. cholerae* O395. Genomic regions unique to the reference strain that are not present in the other strains are without color (no blast hit). The outside numbers (1 to 10 in Ch I and 1 to 13 in ChII), corresponds to the variable regions identified.

A BLAST atlas for the genome of both chromosomes (ChI and ChII) of nine *V. mimicus* and two *V. cholerae* strains were obtained using *V. mimicus* MB451 as the reference strain (Figure 1). The analysis revealed several variable regions in both chromosomes. In ChI, ten major variable regions were detected. Regions 1, 4, 5, 9, and 10 were only present in the reference strain *V. mimicus* MB451, whereas regions 2, 3, 6, 7, and 8 were in most of the *V. mimicus* strains, but were absent in the *V. cholerae* strains. For example, the CDS observed at region 1 were a phage transcriptional regulator AlpA and an integrase (1176 pb), while, in region 4, repeats-in-toxins (RTX) proteins were detected; region 5 consists of the f237 prophage with a zona occludens toxin, accessory cholera enterotoxin, and other CDS of the bacteriophage. Additionally, in region 9, a phage integrase was identified, and, in region 10, putative polysaccharide biosynthesis genes were detected. Differences between the gene content of *V. mimicus* and *V. cholerae* were observed in regions 2, 3, 6, and 8. Such genes as outer membrane proteins, type II/IV secretion system proteins, transcriptional regulators, and a bacteroid aerotolerance operon were identified in *V. mimicus* but not in *V. cholerae*. In region 7, genes, such as chemotaxis proteins, response, and transcriptional regulators, were not present in *V. mimicus* VM573 nor *V. cholerae* strains.

In ChII, thirteen major variable regions were detected. Region 13 and part of region 4 were only present in the reference strain *V. mimicus* MB451. Regions 3, 5, 8, 9, and 10 were present in most *V. mimicus* strains and were absent in the *V. cholerae* strains. Moreover, regions 1, 2, 6, 7, 11, and 12 were absent in most *V. mimicus* strains and in both *V. cholerae* strains. Some of the genes detected only in the reference strain were in region 4, consisting of integral and inner membrane protein genes, DNA damage-inducible genes, Doc toxins, mobile elements, and transcriptional regulators. In region 13, valine glycine repeat G (VgrG) protein genes were observed in the reference strain. In regions 3, 5, 8, 9, and 10, some CDS genes were present in *V. mimicus*, but absent in *V. cholerae*, e.g., outer membrane porin, pilus assembly proteins, type II/IV protein secretion system, and transcriptional regulators. In regions 1, 2, 6, 7, 11, and 12, various genes, such as transcriptional regulators, RTX toxins, and enterotoxins, were absent for most *V. mimicus* strains and *V. cholerae* strains. In addition, a second copy of various flagellar proteins were detected in ChII of *V. mimicus* MB451, VM573, VM603, and SX-4.

As supplementary material, we also included the ANI data of the 21 *V. mimicus* genomes available since the conception of this manuscript to generate the pan-genome atlas (Figure S1), although some of the strains are the same but sequenced by different research groups.

### 3.2. Core and Accessory Genome of V. mimicus

The core genome of *V. mimicus* was obtained by selecting homologous nucleotide sequences with >80% of similarity with the reference genome (*V. mimicus* MB451). Thus, all the positions from the alignment that were not shared with this strain were removed from the core genome analysis. This alignment resulted in 2,972,217 bp for ChI and 1,304,309 for ChII. Next, the core genomes for both chromosomes were built by concatenating the FASTA files of the corresponding aligned core genes, obtaining up to 2,378,529 bp for ChI (2424 core genes) and 826,416 bp for ChII (822 core genes).

**Figure 2:**
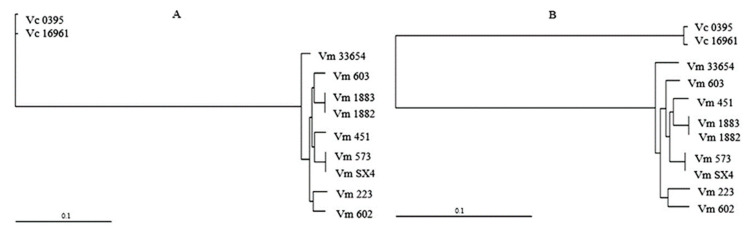
Phylogenetic tree of the core genes of *V. mimicus* MB451, *V. mimicus* VM573, *V. mimicus* SX4, *V. mimicus* CAIM-602^T^, *V. mimicus* ATCC-33654, *V. mimicus* VM603, *V. mimicus* VM223, *V. mimicus* CAIM-1882, *V. mimicus* CAIM-1883, and two *V. cholerae* (Vc O395 and Vc 16961). (**A**): Maximum likelihood (ML) tree of ChI. (**B**): ML tree of ChII.

To determine the phylogenetic relationships of the core genome (CDS) of *V. mimicus* strains, an ML phylogenetic tree was obtained for each chromosome (Figure 2). A clear differentiation of the clades formed by *V. mimicus* and *V. cholerae* was observed. In the phylogenetic trees, a different clustering between *V. mimicus* strains in ChI and ChII was observed, primarily due to the *V. mimicus* MB451 strain. For instance, the environmental *V. mimicus* strains VM603, CAIM-1882, and CAIM-1883 were grouped in ChI but not in ChII, whereas the environmental strains CAIM-1882 and CAIM-1883 clustered with the clinical strain *V. mimicus* MB451. Nonetheless, it can be observed that within the clade of *V. mimicus* the strain ATCC-33654 was the closest to the outgroup in both chromosomes. In addition, species diversity and similar cluster formation were observed (Figure S1).

Furthermore, we obtained genes from the core genome with phylogenetic signal by a likelihood mapping approach. We found 301 genes out of 2196 genes in ChI and 99 genes out of 651 genes in ChII (data not shown). Those genes are considered to have a signal of external origin and could be considered to be the result of recombination and horizontal gene transfer events [22,32]. Virulence genes and transcriptional regulators could be taken as examples of this phylogenetic signal.

**Figure 3:**
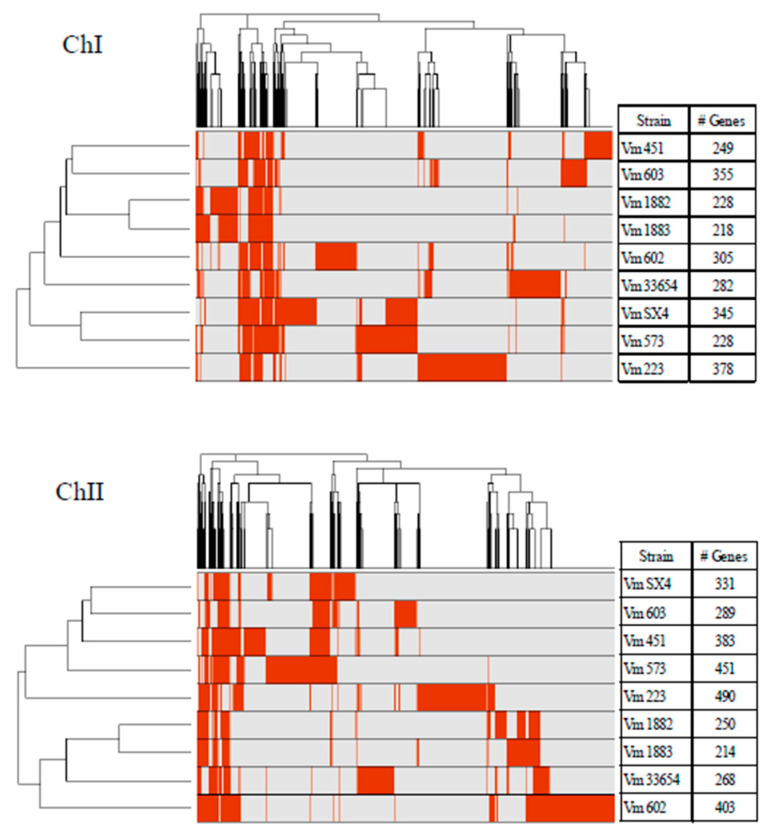
Presence-absence matrix of the genes of the accessory genome of the nine *V. mimicus* strains (*V. mimicus* MB451, *V. mimicus* VM573, *V. mimicus* SX-4, *V. mimicus* CAIM-602^T^, *V. mimicus* ATCC-33654, *V. mimicus* VM603, *V. mimicus* VM223, *V. mimicus* CAIM-1882, and *V. mimicus* CAIM-1883) for each chromosome. Where red color indicates gene presence

For the accessory genome of *V. mimicus*, between 218 and 378 genes were detected in ChI, and for ChII, between 214 and 490 genes were identified (Figure 3), which represents on average 10.9% in ChI and 29% in ChII from the complete genome. The accessory genome was analyzed in terms of the number of genes shared among *V. mimicus* strains. It was observed that most of the genes that were not shared were present only in a single strain or in several. Some genes were present in the nine strains but showed less than 80% of homology; hence, they do not fulfill the criteria for the core genome (Figure S2).

### 3.3. Virulence Classification

**Figure 4:**
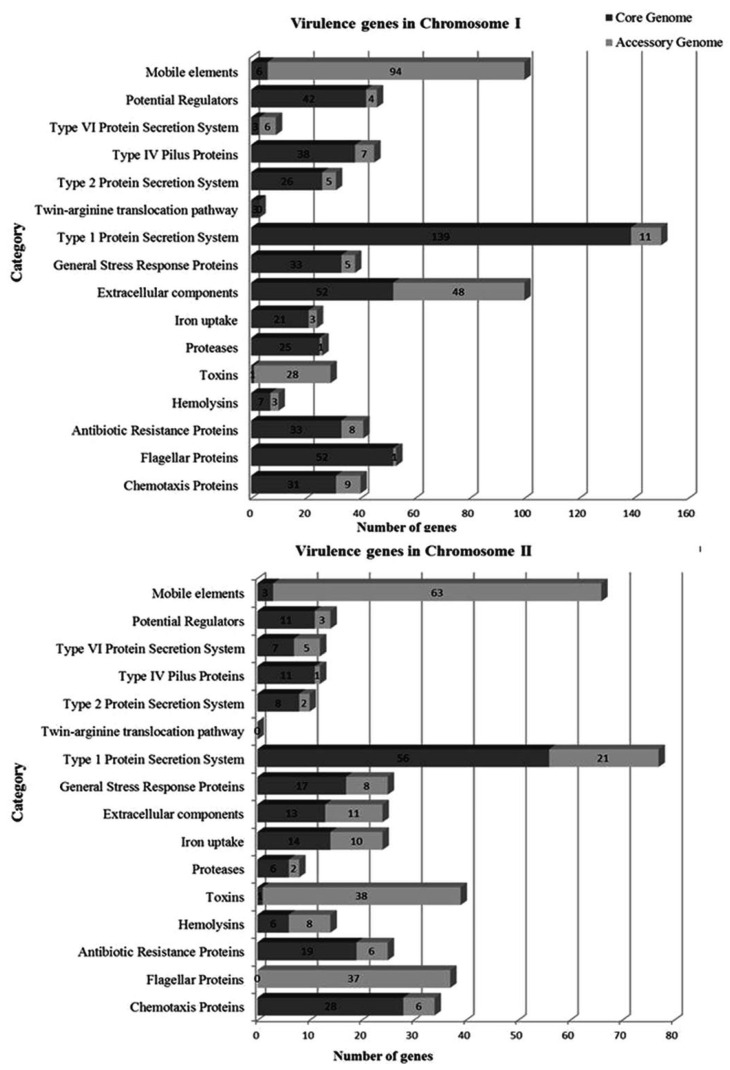
Classification of virulence genes in the core and accessory genome of *V. mimicus* by category.

Virulence genes of the core and accessory genome were identified and classified for each chromosome according to Kimes et al. [41] (Figure 4). In the core genome, 512 virulence genes were identified in ChI and 200 in ChII. The main categories detected in ChI were type I protein secretion system (139 genes), extracellular components (52 genes), flagellar proteins (52 genes), and potential regulators (41 genes). In ChII, the main categories were type I protein secretion system (56 genes), chemotaxis proteins (28 genes), and antibiotic resistance proteins (19 genes). In the accessory genome, 233 virulence genes were identified in ChI and 221 in ChII. The main categories detected in ChI were mobile elements (94 genes), extracellular components (48 genes), and toxins (28 genes). In ChII, the main categories were mobile elements (63 genes), toxins (38 genes), and flagellar proteins (37 genes).

Moreover, virulence genes with phylogenetic signals of the core genome were identified in both chromosomes resulting in 56 genes in ChI and 29 genes in ChII. Phylogenetic trees of some of the virulence genes of ChI (protease, *ompU, mshQ, toxR, luxR, and luxO*) and ChII (*tonB*, zinc metalloprotease, chitinase, *lolC*, methyl-accepting chemotaxis protein, and acriflavine resistance protein) are presented in Figures S3 and S4, respectively. Each phylogenetic tree shows different clusters where *V. mimicus* MB451, *V. mimicus* CAIM-602, *V. mimicus* ATCC-33654, *V. mimicus* VM603, and *V. mimicus* VM223 displayed more variability in each tree of both chromosomes.

### 3.4. MLSA

**Figure 5:**
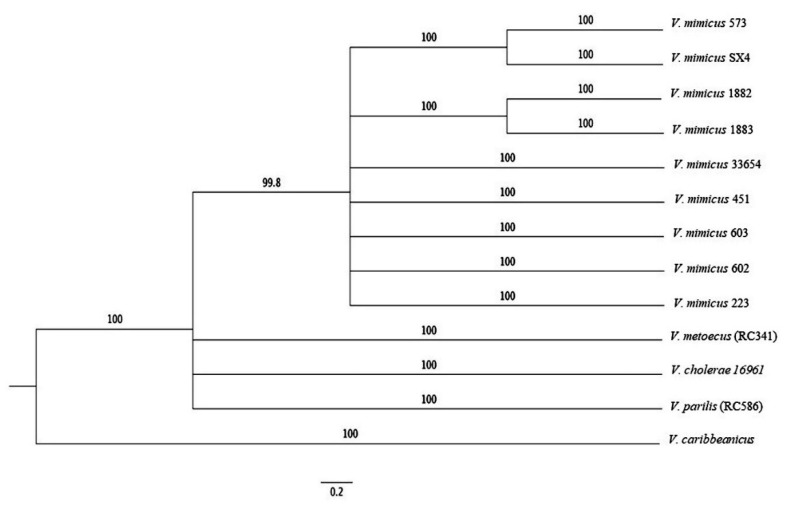
ML phylogenetic tree of eight housekeeping genes *(ftsZ*, *gapA*, *gyrB*, *topA*, *rpoA*, *recA*, *mreB*, and *pyrH*) of the nine strains of *V. mimicus*, *V. cholerae* 16961, *V. parilis (*RC586), *V. metoecus* (RC341), and *V. caribbeanicus* ATCC BAA-2122.

A phylogenetic tree based on MLSA of eight housekeeping genes (*fts*Z, *gap*A, *gyr*B, *top*A, *rpo*A, *rec*A, *mre*B, and *pyr*H) was performed in conjunction with *V. cholerae* 16961, *V. parilis (*RC586), *V. metoecus* (RC341), and *V. caribbeanicus* ATCC-BAA-2122 (Figure 5). The phylogenetic trees assigned the analyzed species to a different clade, where *V. mimicus* forms a clearly separated clade from the others. The clusters observed provide evidence of the differentiation of *V. mimicus* strains with other *Vibrio* species and its variability.

## 4. Discussion

Comparative genomics analyses have been performed for years, primarily with partial genomes. Due to advances in technology and accessibility, increasingly complete bacterial genomes are now available. Hence, it is possible to identify all the genes present in a specific genome [42]. However, at the time of this study, only nine genomes sequences of *V. mimicus* were available, including one obtained by our research group that was uploaded in 2019 (Accession: PRJNAV.231624). By September 2019, twenty-one assembled genomes were available at NBCI, and this number is still increasing. A pan-genome atlas, including all 21 *V. mimicus* genomes, was constructed, although part of the strain’s information is missing. In addition, some of the available genomes correspond to the same strain but sequenced by different research groups (e.g., CAIM602 and NCTC11435; FDAARGOS_112 and ATCC 33654), and there were slight differences and a different number of unique genes observed. Probably, those differences could be due to bacterial subculturing along time by each research group, which can lead to the selection of different variants carrying a distinct set of mutations [43]. Differences in storage and preservation methods, and general laboratory practices, may also contribute to this phenomenon. Moreover, some strains were isolated from the same region, year, and clustered together (Figure S1). Therefore, a reduced number of strains, including all the genome and metadata, were selected for genome comparison. Despite the low number of genomes of *V. mimicus* included, this work presents the first comparative genome analysis of *V. mimicus*, obtaining a first outline of the pan-genome, core genome, and accessory genome.

Quantification of genome relatedness can be accomplished by different methods [44]. The ML phylogenetic trees were obtained for the core genome of *V. mimicus*. These phylogenetic trees showed no correlations among strains relatedness and its source (clinical or environmental), isolation year, or geographical origin. Surprisingly, only a few strains clustered together based on its source, and two clinical (573 and SX4) and two environmental (1882 and 1883) strains showed consistency between them in the phylogenetic trees. Nevertheless, it is important to note the major lapse between strains and the distant geographical areas from which they were isolated, which may help to explain the lack of correlation.

Eight housekeeping genes were used to study the relationships between *V. mimicus* and three closely related species, *V. cholerae*, *V. parilis (*RC586), and *V. metoecus (*RC341) using *V. caribbeanicus* as an outgroup. A separate clade formed by *V. mimicus* strains was observed, corroborating the differences with *V. cholerae* and the others. Surprisingly, *V. cholerae* was more closely related to the two other species than *V. mimicus*. Sawabe et al. [45] realized MLSA with the same eight housekeeping genes in several *Vibrio* species. These researchers identified a cholerae clade that includes *V. cincinnatiensis*, *V. cholerae, V. furnissii*, *V. fluvialis*, *V. metschnikovii*, *V. cholerae*, and *V. mimicus*, and the similarity of the MLSA concatenation was between 85.4% and 94.7% within this clade. Recently, an update of the *Vibrio* clades was published, and *V. parilis* was added to the cholera clade with almost the same similarity values (83.4–94.4%) [46]. The MLSA of *V. mimicus* species shows in the ML tree consistency in their clustering. Thus, this approach is useful for studying phylogenetic relationships, and MLSA offers a good option because fewer data and less time are required for the analysis.

Phylogenetic signals within the core genome genes were observed, although there was a small number of positive genes (301 genes in ChI and 99 genes in ChII). As the core genome includes genes sharing >80% homology in the sequence, it is unsurprising to detect few differences between them, which may help to explain the low number of genes showing phylogenetic signals. However, these genes with phylogenetic signals could be considered to have a different phylogenetic history and therefore could be potentially recombinant (either by recombination or horizontal gene transfer) [32]. In the accessory genome analysis, strong variability was observed between the strains; different numbers of genes were reported in each strain, and an aggrupation between strains was made depending on the number of strains that shared the same gene. These results help us to elucidate the variability of the species.

The development of molecular and genomic techniques allows the identification of genes that encode virulence factors [47]. Although *V. mimicus* and *V. cholerae* are closely related and share some phenotypic and genomic characteristics, differences in the genome Blast atlas have been reported [3,6,9]. The main differences found in this study were on virulence genes, hypothetical proteins, and transcriptional regulators. These differences in virulence content could explain the distinctiveness of the pathogenic potential of each species [48]. Several virulence genes were identified and classified in this study for *V. mimicus* strains. More virulence genes were found in the core-genome, with a higher number in ChI (511 genes) than in ChII (200 genes), the same pattern, but with lower values were observed in the accessory genome (233 genes in ChI and 221 genes in ChII). It has been reported that ChI has more plasticity [8] and more virulence genes than ChII. The genes present in ChI are responsible for growth and viability, while the genes in ChII are responsible for adaptation to environmental changes [1,6]. However, in *V. cholerae*, it has been proposed that the genes on both chromosomes function differently depending on the environment [49]. Thus, it would be important for future research to study how the gene content of *V. mimicus* could depend or be affected by the environment.

## 5. Conclusions

With the advances and development of biological technologies, information concerning bacterial evolution and pathogenesis has become increasingly available. The results obtained in this study regarding the pan-genome (core genome, accessory genome, and virulence genes), as well as phylogenetic analysis of *V. mimicus*, will help us to elucidate the variability of this species. These results, even in cases when they came from the same source, have provided a perspective on the gene content and virulence potential of *V. mimicus*. A great diversity of genes associated with virulence factors, previously linked to other pathogenic *Vibrio* species, were found in the core and accessory genomes of *V. mimicus*. This finding emphasizes the pathogenic potential of *Vibrio mimicus*, as well as the importance of continuing with the genomic study of this species. Specifically, further research is warranted to elucidate the pathogenic potential and its possible mechanisms, as well as to establish the behavior of *V. mimicus* in the environment and evaluate its real potential as a human pathogen.

## Figures and Tables

**Table 1:** General characteristics of the nine genomes of *V. mimicus* included in this study.

	*V. mimicus*								
	CAIM 602^T^	ATCC 33654	CAIM 1882	CAIM 1883	VM MB451	VM223	VM573	VM603	SX-4
Source	Clinical	Environmental	Environmental	Environmental	Clinical	Environmental	Clinical	Environmental	Clinical
Origin	Ear infection	River water	Shrimp process water	Shrimp process water	Diarrhoea	Bivalve	Diarrhoea	Fluvial water	Diarrhoea
Country	North Carolina, EU	Louisiana, EU	Guaymas, México	Guaymas, México	Shanxi, Chin	Sao Paulo, Brazil	EU	Amazonia, Brazil	China
Year	80s	80s	2012	2012	2009	-	90s	90s	2009
Size Chr1 (bp)	2,934,158	2,938,455	2,819,391	2,820,150	2,972,217	3,055,543	2,880,536	2,894,575	2,997,127
No. subsystems	434	434	427	426	436	434	415	434	435
No. CDS	2729	2706	2446	2642	2673	2802	2652	2779	2769
No. RNAs	87	85	79	77	115	107	75	49	91
% GC	46.6	46.6	46.8	46.8	46.6	46.4	46.3	46.6	46.4
Size Chr2 (bp)	1,268,270	1,191,392	1,141,600	1,115,258	1,304,309	1,292,428	1,460,636	1,247,610	1,276,483
No. subsystems	112	113	110	109	114	108	132	111	113
No. CDS	1225	1090	1072	1036	1205	1312	1273	1111	1153
No. RNAs	6	4	5	6	4	4	18	4	4
% GC	45.9	46.4	46.5	46.5	45.7	45.7	45.8	45.8	45.8
Size Genome	4,202,428	4,129,847	3,960,991	3,935,408	4,276,526	4,347,971	4,341,172	4,142,185	4,273,610
Total Genes	3954	3796	3518	3678	3878	4114	3925	3890	3922

## Data Availability

The data presented in this study will be openly available at CIAD thesis repository (https://ciad.repositorioinstitucional.mx/jspui/) from 2021.

## Supplementary Materials

The following are available online at https://www.mdpi.com/2076-2607/9/1/191/s1, Figure S1. Pan-genome atlas of 21 *V. mimicus strains: V. mimicus* MB451 *V. mimicus* VM573, *V. mimicus* SX-4, *V. mimicus* CAIM-602^T^, *V. mimicus* ATCC-33654, *V. mimicus* VM603, *V. mimicus* VM223, *V. mimicus* CAIM-1882, *V. mimicus* CAIM-1883, *V. mimicus* FDAARGOS113, *V. mimicus* FDAARGOS112, *V. mimicus* 523-80, *V. mimicus* NCTC11435, *V. mimicus* N2733, *V. mimicus* N2763, *V. mimicus* N2781, *V. mimicus* N2789, *V. mimicus* N2790, *V. mimicus* N2810, *V. mimicus* N2816, and *V. mimicus* SCCF01. Figure S2. Classification of the accessory genome of *V. mimicus* according to the number of shared genes between strains. Figure S3. Phylogenetic tree of six virulence genes with phylogenetic signal (protease, *ompU*, *mshQ*, *toxR*, *luxR*, and *luxO*) in ChI of *V. mimicus* MB451, *V. mimicus* VM573, *V. mimicus* VM603, *V. mimicus* SX4, *V. mimicus* CAIM 602^T^, *V. mimicus* ATCC 33654, *V. mimicus* VM223, *V. mimicus* CAIM 1882 and *V. mimicus* CAIM 1883; using *V. cholerae* 16961 and *V. cholerae* O395 as outgroups. The phylogenetic tree was obtained by an independent maximum likelihood (ML) tree reconstruction using RAxML v7.2.7 [25] and the topology testing methodology implemented in TreePuzzle v5.2 [32]. Figure S4. Phylogenetic tree of six virulence genes with phylogenetic signal (*ton*B, zinc metalloprotease, chitinase, *lol*C, methyl.accepting chemotaxis protein, and acriflavine resistance protein) in ChII of *V. mimicus* MB451, *V. mimicus* VM573, *V. mimicus* VM603, *V. mimicus* SX4, *V. mimicus* CAIM 602^T^, *V. mimicus* ATCC 33654, *V. mimicus* VM223, *V. mimicus* CAIM 1882 and *V. mimicus* CAIM 1883; using *V. cholerae* 16961 and *V. cholerae* O395 as outgroups. The phylogenetic tree was obtained by an independent maximum likelihood (ML) tree reconstruction using RAxML v7.2.7 [25] and the topology testing methodology implemented in TreePuzzle v5.2 [32].
