# Supplementary material for: Core and Accessory Genome Analysis of Vibrio mimicus
Source: Microorganisms. 2021 Jan 18;9(1):191. doi: 10.3390/microorganisms9010191 (PMC7831076; doi:10.3390/microorganisms9010191)
Supplement: Supplementary file 1 [file microorganisms-09-00191-s001.pdf]

Supplementary Material

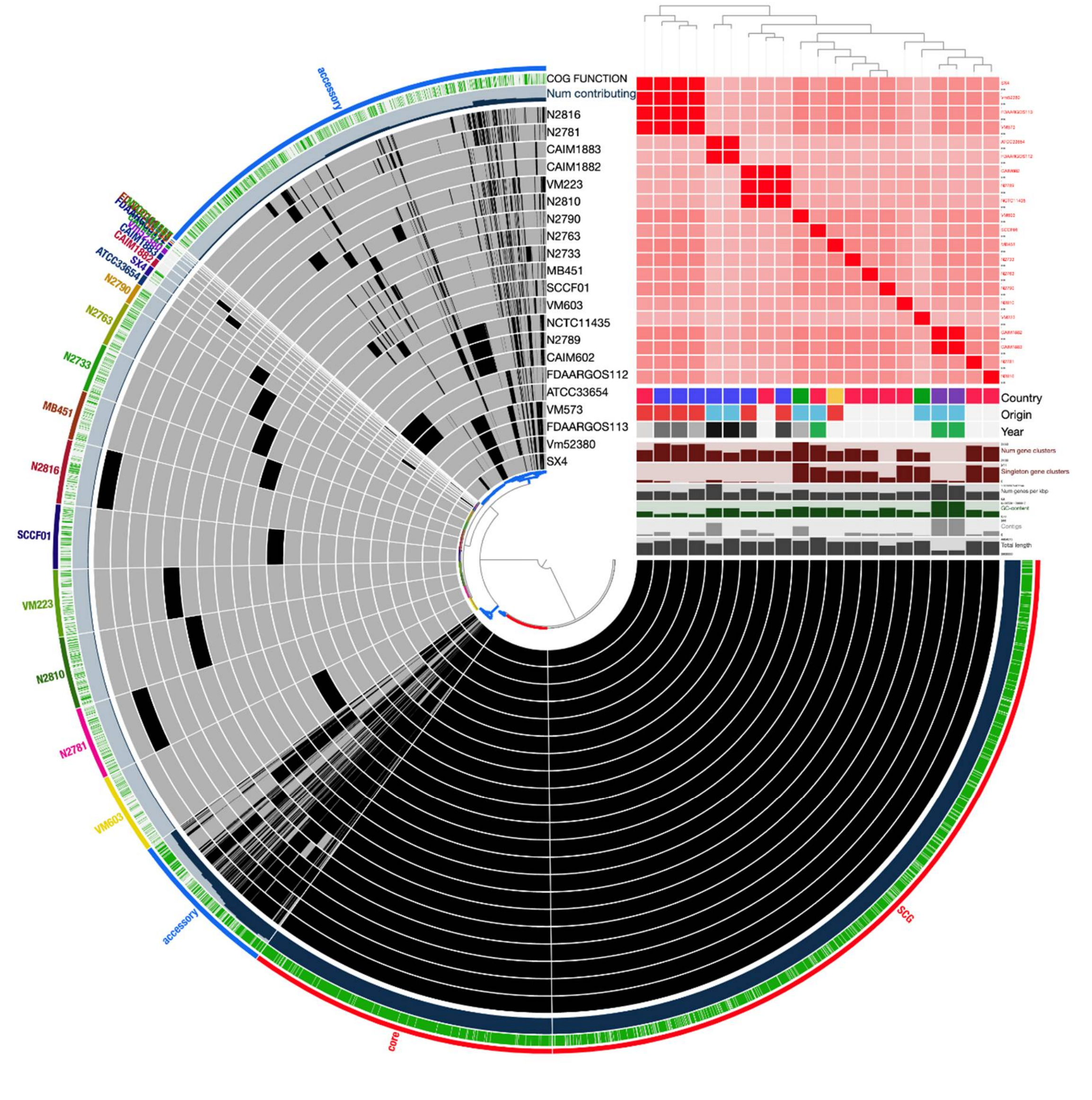

S1. Pan-genome atlas of 21 *V. mimicus strains: V. mimicus* MB451 *V. mimicus* VM573, *V. mimicus* SX-4, *V. mimicus* CAIM-602^{T}, *V. mimicus* ATCC-33654, *V. mimicus* VM603, *V. mimicus* VM223, *V. mimicus* CAIM-1882, *V. mimicus* CAIM-1883, *V. mimicus* FDAARGOS113, *V. mimicus* FDAARGOS112, *V. mimicus* 523-80, *V. mimicus* NCTC11435, *V. mimicus* N2733, *V. mimicus* N2763, *V. mimicus* N2781, *V. mimicus* N2789, *V. mimicus* N2790, *V. mimicus* N2810, *V. mimicus* N2816, and *V. mimicus* SCCF01

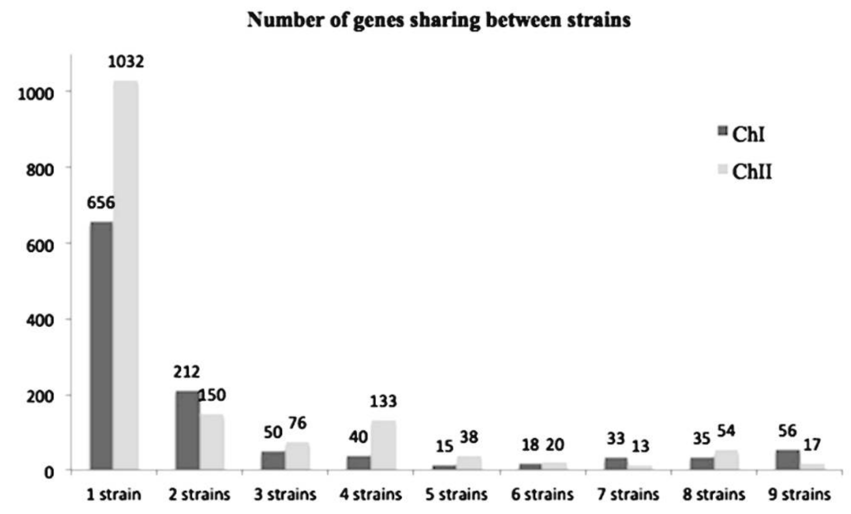

Figure S2. Classification of the accessory genome of *V. mimicus* according to the number of shared genes between strains

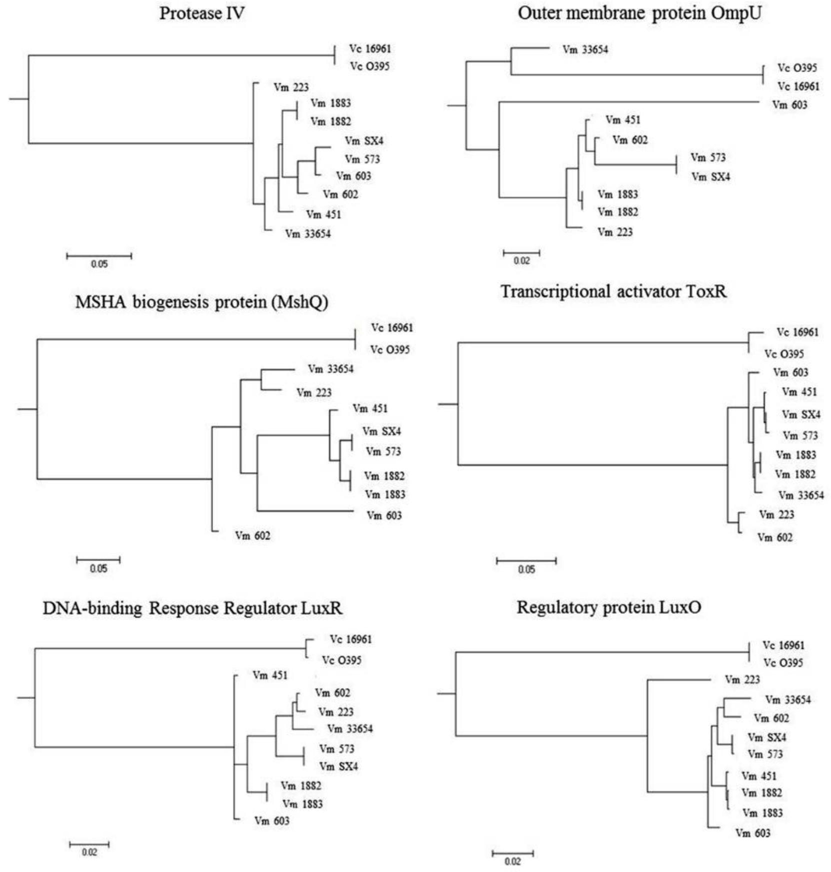

Figure S3. Phylogenetic tree of six virulence genes with phylogenetic signal (protease, *ompU*, *mshQ*, *toxR*, l*uxR*, and *luxO*) in ChI of *V. mimicus* MB451, *V. mimicus* VM573, *V. mimicus* VM603, *V. mimicus* SX4, *V. mimicus* CAIM 602^{T}, *V. mimicus* ATCC 33654, *V. mimicus* VM223, *V. mimicus* CAIM 1882 and *V. mimicus* CAIM 1883; using *V. cholerae* 16961 and *V. cholerae* O395 as outgroups. The phylogenetic tree was obtained by an independent maximum likelihood (ML) tree reconstruction using RAxML v7.2.7 [25] and the topology testing methodology implemented in TreePuzzle v5.2 [32].

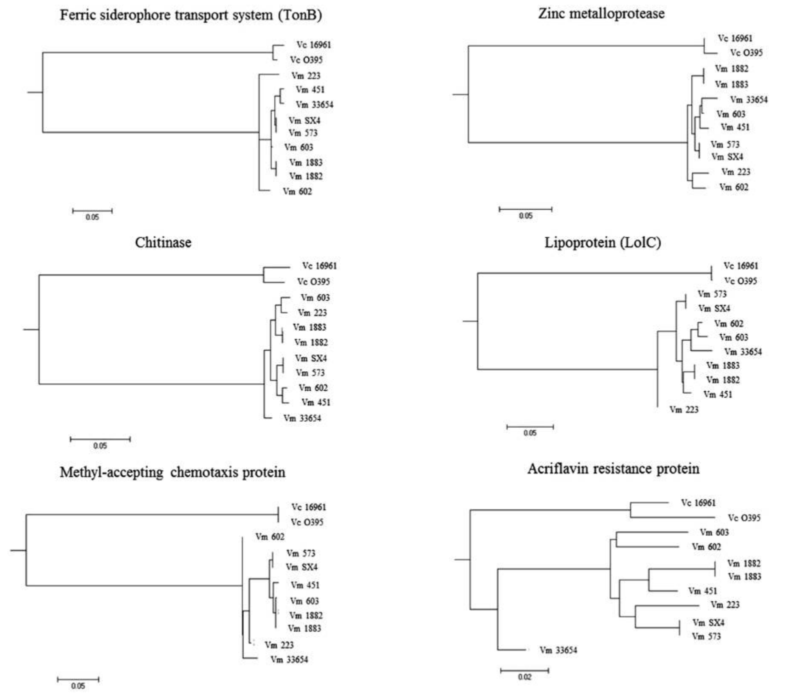

Figure S4. Phylogenetic tree of six virulence genes with phylogenetic signal (*ton*B, zinc metalloprotease, chitinase, *lol*C, methyl.accepting chemotaxis protein, and acriflavine resistance protein) in ChII of *V. mimicus* MB451, *V. mimicus* VM573, *V. mimicus* VM603, *V. mimicus* SX4, *V. mimicus* CAIM 602^{T}, *V. mimicus* ATCC 33654, *V. mimicus* VM223, *V. mimicus* CAIM 1882 and *V. mimicus* CAIM 1883; using *V. cholerae* 16961 and *V. cholerae* O395 as outgroups. The phylogenetic tree was obtained by an independent maximum likelihood (ML) tree reconstruction using RAxML v7.2.7 [25] and the topology testing methodology implemented in TreePuzzle v5.2 [32].
